# A critical role for the self-assembly of Amyloid-β1-42 in neurodegeneration

**DOI:** 10.1038/srep30182

**Published:** 2016-07-22

**Authors:** Karen E. Marshall, Devkee M. Vadukul, Liza Dahal, Alina Theisen, Milena W. Fowler, Youssra Al-Hilaly, Lenzie Ford, György Kemenes, Iain J. Day, Kevin Staras, Louise C. Serpell

**Affiliations:** 1 School of Life Sciences, University of Sussex, Falmer, BN1 9QG, UK; 2 College of Sciences, Chemistry Department, Al-Mustansiriyah University, Baghdad, Iraq

## Abstract

Amyloid β1-42 (Aβ1-42) plays a central role in Alzheimer’s disease. The link between structure, assembly and neuronal toxicity of this peptide is of major current interest but still poorly defined. Here, we explored this relationship by rationally designing a variant form of Aβ1-42 (vAβ1-42) differing in only two amino acids. Unlike Aβ1-42, we found that the variant does not self-assemble, nor is it toxic to neuronal cells. Moreover, while Aβ1-42 oligomers impact on synaptic function, vAβ1-42 does not. In a living animal model system we demonstrate that only Aβ1-42 leads to memory deficits. Our findings underline a key role for peptide sequence in the ability to assemble and form toxic structures. Furthermore, our non-toxic variant satisfies an unmet demand for a closely related control peptide for Aβ1-42 cellular studies of disease pathology, offering a new opportunity to decipher the mechanisms that accompany Aβ1-42-induced toxicity leading to neurodegeneration.

Alzheimer’s disease (AD) is characterised by the deposition of Aβ as amyloid fibrils in extracellular plaques, and the intracellular accumulation of tau as neurofibrillary tangles in the brain. Mutations in Aβ and the amyloid precursor protein (APP) are linked to familial AD; hence Aβ is thought to play an important role in the disease process^1^,^2^. Numerous studies have been carried out to try to better understand how Aβ contributes to the neurodegeneration observed in AD patients and the symptoms of the disease. Self-assembled Aβ has been shown to cause membrane defects^3^,^4^, disruption to neuronal networks^5^,^6^, neuronal dysfunction^7^,^8^,^9^, impairment of long-term potentiation^10^, and changes in animal behavior^7^,^11^,^12^. Based on these reports, the current consensus is that Aβ conversion and self-assembly into oligomeric forms is responsible for neuronal death in AD, although the exact role of fibrillar amyloid forms of Aβ and the specific mechanisms of toxicity are still very much debated.

The Aβ peptide is a member of a large group of amyloidogenic peptides and proteins^13^ whose toxic properties are believed to be specifically linked to their amyloidogenicity^14^. However, a detailed mechanistic understanding of how Aβ misfolding leads to neuronal dysfunction and eventual death remains limited. One critical constraint has been the lack of a suitable peptide against which the toxic properties of Aβ can be rigorously assessed. At present, most researchers typically use a buffer or alternative vehicle solution, but neither takes account of the structural features of Aβ oligomers or other aggregated isoforms that may be responsible for neurotoxic actions. Although scrambled, reversed or rodent Aβ1-42 sequences are occasionally used as controls, the assembly, structure and toxicity of these peptides have never been characterized in detail. Therefore a non-assembling peptide is not available to compare the effects with Aβ1-42. In practise, a peptide with an essentially identical size and sequence to Aβ is required, but which neither assembles nor causes cell death. The importance of using assembly-incompetent peptides cannot be understated if robust conclusions are to be drawn on how self-assembly and amyloidogenesis contributes to disease. A second issue that has hampered progress in delineating Aβ action, is the propensity of the peptide to self-assemble: a process that is difficult to control since it is influenced by many factors such as peptide concentration, solvent type, temperature and the presence of seeds that can accelerate assembly^15^,^16^. As a result, preparation procedures are liable to substantial variability in the type and size of aggregates formed^16^,^17^ which confounds simple interpretation of findings into what species contribute to cell death. Importantly, although monomeric Aβ has not been implicated as playing a role in toxic effects, it has not been previously possible to ensure the preparation of pure monomeric Aβ, and that it remains so for extended periods. For the first time, we have rationally designed and extensively characterized a non-oligomeric peptide, representing a major breakthough in the ability to dissect which structural species are responsible for Alzheimer’s deficits.

Here, we report an approach that addresses two major limitations to AD research; the lack of a non-assembling peptide to compare to, and the need for a preparation method that produces consistent oligomer populations, and provides the basis for tightly-controlled studies of Aβ action. Specifically, we outline the rational design and generation of a non-aggregation prone variant of Aβ, identical to the wild type 1–42 sequence with the exception of two amino acid substitutions. We show that in combination with a protocol designed to remove pre-aggregates and potentially contaminating solvents^3^,^17^ we can produce highly consistent peptide preparations of Aβ1-42 and compare it to vAβ1-42 prepared under identical conditions. To gain insights into how the amyloidogenicity of Aβ1-42 impacts on its effects, we carried out a full structural and functional characterisation of both peptides. Compared to wild-type Aβ, we show that vAβ1-42 is compromised in its ability to aggregate and thus rendered non-toxic. Even over extended periods (7 days), we find that the variant peptide does not assemble into oligomers or fibres or develop any characteristic β-sheet structure. Furthermore, unlike Aβ1-42 oligomers, vAβ1-42 is not detected associated with or internalised into primary hippocampal neurons and has no effect on the metabolic activity or viability of neuroblastoma cells or neurons. Using optical imaging approaches we show that presynaptic viability and vesicle recycling is compromised in Aβ treated but not vAβ-treated neurons. Using a whole animal model system, which is routinely employed for studying behavioural learning^18^, we show that normal memory formation occurs in vAβ1-42 but not Aβ-treated animals. This peptide illustrates the key role of amino acid sequence in the ability of Aβ1-42 to assemble and form toxic structures. Furthermore it can act as a robust control, providing an indispensible tool for studies on Aβ in AD.

## Results and Discussion

### Rational design of a non-assembling variant of the Aβ1-42 peptide

**Figure 1:**
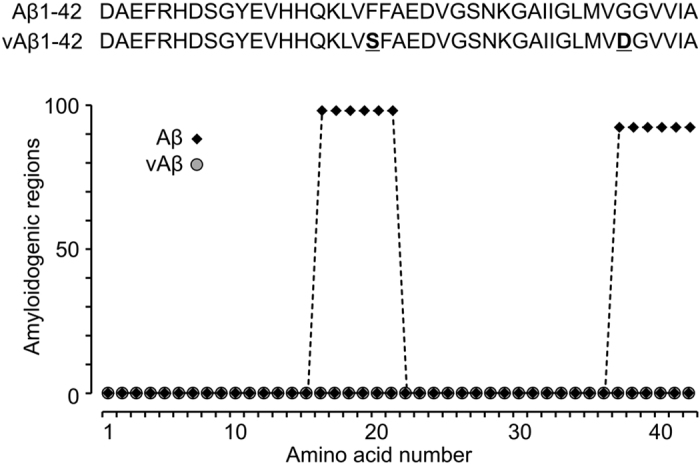
The graph produced using WALTZ^19^ shows two peaks that indicate two amyloidogenic regions (residues 16–21 and residues 37–42) in Aβ1-42 compared to the trace for vAβ1-42 peptide design showing abolition of the amyloidogenic regions.

WALTZ algorithm^19^ was used to predict the effects of amino acid substitutions on the propensity of wildtype (WT) Aβ1-42 to form amyloid. WALTZ identifies two regions of WT Aβ1-42, between 15–22 and 36–42 that have the most amyloid-forming potential (Fig. 1). All possible amino acid substitutions were introduced into these regions and examined for amyloidogenicity using WALTZ^19^. The results were ranked and those substitutions that result in abolition of amyloidogenicity peaks were examined further. Substituting phenylalanine at position 19 (F19) with serine (S) and glycine at position 37 (G37) with aspartic acid (D) results in removal of the amyloidogenic regions in the Aβ1-42 peptide (Fig. 1). Previous studies have shown that phenylalanine and F19 in particular in Aβ plays an important role in driving assembly^20^,^21^,^22^ and solid state NMR measurements of Aβ specifically show molecular contacts between F19 and L34^23^,^24^. Single substitutions of F19 have shown a significant reduction in Aβ assembly^21^,^22^. Substitution of F19 for leucine revealed that the F19 residue plays an important role in nucleation^25^. Glycine zippers have also been implicated in the amyloidogenic process and oligomer toxicity^26^. Aβ1-42 with G37L substitution dramatically reduced the toxic effects in cell culture and *in vivo* in *Caenorhabditis elegans*^26^. NMR studies have revealed that G37 fits into a solvent accessible turn together with G38 within oligomers^24^. Therefore, G37D was selected to disrupt this architecture. The substitution of hydrophobic F19 for smaller, polar serine and G37, for the negatively charged, bulky aspartic acid was predicted to disrupt the intermolecular contacts that drive and stabilise assembly into oligomers and fibrils.

### F19S G37D substitutions impair assembly of Aβ1-42

Aβ1-42 F19S G37D, hereafter referred to as vAβ1-42, was characterised alongside WT Aβ1-42 using a range of biophysical techniques that report on the structural changes that take place during assembly. The peptides were prepared using a method that ensures removal of preformed aggregates and any potentially contaminating solvents as described in detail in the methods^3^,^17^. Peptide concentration was measured immediately after preparation and then the solution diluted to 50 μM to ensure reproducibility between experiments. We followed the assembly of both peptides over the course of one week using transmission electron microscopy (TEM) as well as spectroscopic and immunological methods to fully examine their assembly properties.

**Figure 2:**
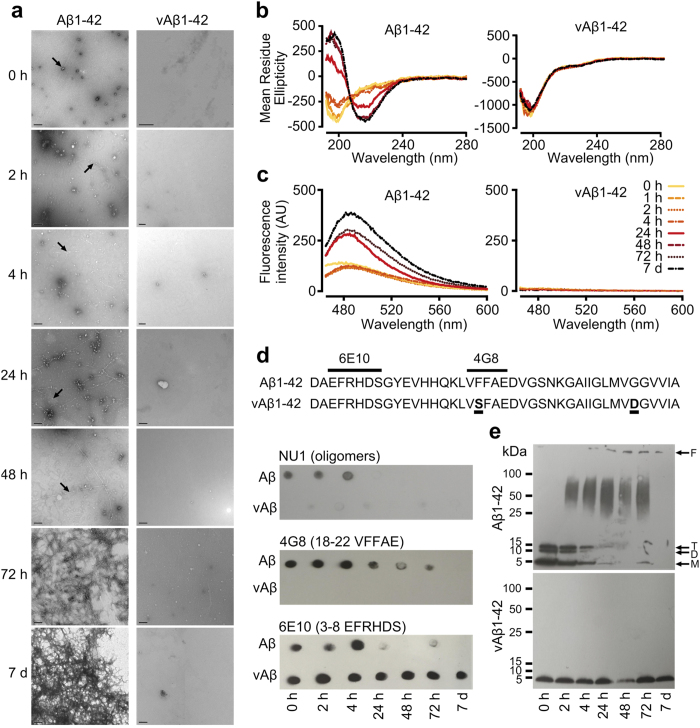
(**a**) Negative stain transmission electron microscopy images of Aβ1-42 (left panels) and vAβ1-42 peptide (right panels) showing assembly of Aβ1-42 into fibrils at around 24 hours, preceded by small spherical structures (both shown by arrows). Conversely the vAβ1-42 peptide does not form fibres even up to 7 days. All peptides were prepared as described in the methods at 50 μM. Scale bars 200 nm. (**b**) CD spectra of Aβ1-42 and vAβ1-42 over time showing the formation of β-sheet structures for Aβ1-42 after around 24 hours whilst vAβ1-42 remains as random coil structure up to the final time point of 7 days. (**c**) Thioflavin T fluorescence showing increasing fluorescence at 483 nm of Aβ1-42 over 7 days, compared to no change in fluorescence of vAβ1-42. (**d**) Sequence of Aβ1-42 (top) and vAβ1-42 (bottom), showing epitope regions for 4G8 and 6E10 antibodies. The amino acid substitutions are underlined. Dot blots using anti-oligomer antibody, NU1 and anti-Aβ antibodies 4G8 and 6E10 show oligomer reactive species only in Aβ1-42 samples and not vAβ1-42. Similarly, 4G8 does not detect vAβ1-42 due to the F19S substitution in the epitope region. 6E10 binds both Aβ1-42 and vAβ1-42 as the epitope is the same in both peptides. (**e)** Western blot of Aβ1-42 (top) and vAβ1-42 (bottom) with 6E10 over time shows monomers (M), dimers (D), trimers (T), higher molecular weight species and fibres (F) are only formed by the wild-type peptide. vAβ1-42 runs as a monomer.

The morphology of the structures was visualised over time using TEM (Fig. 2a). Even at very early time points small spherical assemblies could be observed in the Aβ1-42 sample (Fig. 2a), consistent with previous reports^3^,^27^ whilst the vAβ1-42 sample was free from any aggregates. After 2 hours, very small worm-like structures appear in Aβ1-42, which resemble protofibrils (Fig. 2a). After 24 hours long fibrils form and are present in abundance by 72 hours. These are similar to those shown in recent studies^27^,^28^,^29^. Conversely, vAβ1-42 does not form fibrils, even up to one week after preparation.

To further investigate if vAβ1-42 was undergoing any structural reorganisation that could not be detected using TEM, temporal changes in the secondary structure of the peptide were monitored using circular dichroism (CD) (Fig. 2b). CD spectra confirm that Aβ1-42 is initially random coil for two to four hours (minima at 200 nm) and converts to β-sheet conformation (maximum at 195 nm and minimum at 218 nm). After 24 hours a clear β-sheet signal is apparent that becomes stronger at 48 hours, concomitant with the appearance of fibres by TEM. vAβ1-42 remains in a random coil conformation for the duration of the experiment (7 days). This confirms that the vAβ1-42 peptide remains soluble and does not assemble into a β-sheet rich structure.

To examine the fibrillogenesis process, a Thioflavin T (ThT) fluorescence assay was carried out to monitor amyloid assembly with time (Fig. 2c). Aβ1-42 shows some fluorescence intensity even at early time points, indicating the presence of small species able to bind the dye. However this signal significantly increases at 24 hours, when fibres are visible by TEM and when a β-sheet CD signal predominates. vAβ1-42 shows negligible intensity at 483 nm. Together these biophysical data suggest that vAβ1-42 does not self-assemble into amyloid while wild-type Aβ1-42 forms small oligomeric species followed by β-sheet rich amyloid fibres.

Solution state NMR has been used to monitor the change in solubility of Aβ peptides over time^30^, since the signal arising from the soluble peptide disappears as the peptide assembles beyond a certain size. Here, we examined the 1H NMR spectra from vAβ1-42 over three days and confirmed that the NMR signal remained strong and identical to time zero indicating that the peptide does not change structure or self-assemble during this time (Supplementary Figure 1).

### vAβ1-42 does not form oligomers

Although the data outlined above indicate that vAβ1-42 is not assembling into species that may contain β-sheet structure, it is possible that it is able to assemble into very small oligomers of a size below the resolution of TEM, and that do not exhibit β-sheet conformation or bind ThT. To explore this further, we used a series of antibodies directed against different conformations and epitopes to determine whether vAβ1-42 was forming any assemblies resembling those formed by Aβ1-42. Antibodies that bind to Aβ oligomers (NU1)^31^ or raised against the N or C-terminus (6E10 and 4G8 respectively) of Aβ were used for dot blotting (Fig. 2d). Aβ1-42 and vAβ1-42 were prepared at the same time points as in Fig. 2a–c. The oligomer-specific antibody (NU1) detected only Aβ1-42 indicating that vAβ1-42 does not form antibody detectable oligomers. The N-terminal antibody, 4G8, does not bind to vAβ1-42 as expected since the epitope overlaps with the F19S substitution. In contrast 6E10 binds to both peptides, confirming that vAβ1-42 can be detected by dot blotting. Notably there is a decrease in signal for each antibody with Aβ1-42 incubation time. This is consistent with changes in conformation that accompany assembly to form fibrils which would reduce available epitopes. In contrast, there is no change in 6E10 intensity for vAβ1-42 over time consistent with the peptide remaining monomeric for up to 7 days.

Although dot blotting provides a useful way of looking at the antigenicity of Aβ1-42 and vAβ, due to the dynamic nature of amyloid assembly, the samples applied to the membrane probably contain a heterogeneous mixture of different species. To detect any assembled structures, we ran SDS-PAGE using samples collected at different incubation time points and then carried out a western blot with 6E10 (Fig. 2e). At the start of the experiment Aβ1-42 is predominantly in a monomeric conformation (M) and migrates at around 4.5 kDa, with a significant population of dimers (D, ~9 kDa) and trimers (T, ~13.5 kDa) present. After 2 hours, higher molecular weight oligomers form. These species migrate as a smear and, at later time points, the lower molecular weight monomer and oligomers disappear. After 7 days there are no oligomeric species present at all and fibrils are observed in the well of the gel. vAβ1-42 remains monomeric and migrates at 4.5 kDa for each incubation time point in the experiment (Fig. 2e).

### vAβ1-42 is not toxic to primary hippocampal neurons and has no effect on metabolic activity in a human neuroblastoma cell line

Having established that in contrast to Aβ1-42, vAβ1-42 does not assemble into oligomers or fibrils, we sought to determine if there was a corresponding difference in function. There are numerous reports suggesting that pre-fibrillar, oligomeric species are the cause of the neurodegeneration observed in AD and toxicity in cell culture^7^,^8^. The preceding data reveals that Aβ1-42 is mostly oligomeric (i.e. not fibrillar, lacking in β-sheet structure and identifiable by anti-oligomer antibodies) at 2 to 4 hours post preparation. Therefore, for the remaining experiments, the peptide preparations were used at this time point. In addition, we chose to carry out these experiments out at a final peptide concentration of 10 μM, to ensure that any negative results were not due to low levels of peptide and accelerate the peptide effects within a reasonable time-frame.

**Figure 3:**
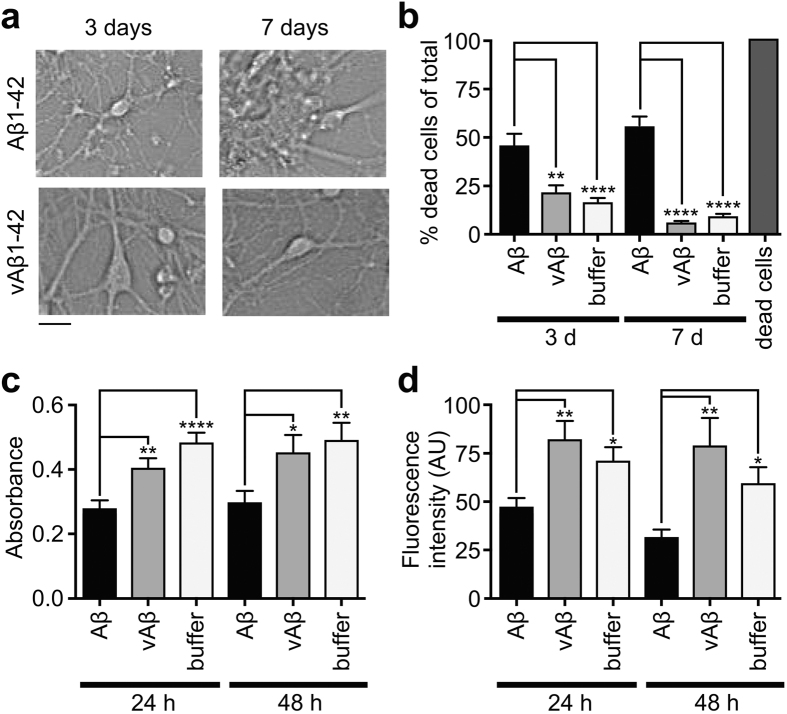
(**a**) DIC widefield images of neurons live in culture following treatment with either Aβ1-42 oligomers or vAβ1-42 after 3 or 7 days. Some live neurons are still clearly visible in the Aβ1-42 culture after 3 days but by 7 days none appear healthy. Scale bar 20 μm. (**b**) Measurement of proportion of dead cells compared to the total counted in culture by Readyprobes assay following 3 and 7 days exposure to Aβ1-42 or vAβ1-42 or buffer only (total number of cells counted (number of dead cells in brackets) at 3 days: n = 1163 (545), 1012 (220) and 1661 (279) and 7 days: n = 1032 (610), 684 (37) and 1112 (97) for Aβ1-42, vAβ1-42 and buffer respectively). (**c**) MTT assay (24 hours: n = 19, 9 and 20, 48 hours: n = 14, 9 and 13 for Aβ1-42, vAβ1-42 and buffer respectively) and (**d**) CTB (24 hours: n = 12, 11, and 16, 48 hours: n = 12, 9, and 18 for Aβ1-42, vAβ1-42 and buffer respectively) assay using SH-SY5Y cells. 10 μM oligomeric Aβ1-42 has a significant effect on the cells after 24 hours whilst vAβ1-42 is the same as buffer only. Unpaired parametric student’s t test, only significant differences are shown, where p = < 0.05 (*), <0.01 (**), <0.0001 (****) and >0.05 was not significant. Error bars are expressed as ±SEM.

Aβ1-42 oligomers have been shown to be toxic to cultured neuroblastoma cells and neurons^27^,^32^. In order to investigate this effect of Aβ1-42 on cells and compare to vAβ1-42, we conducted cell viability assays on two different cell types, shown in Fig. 3. First, a live/dead cell assay was carried out using primary rat hippocampal neurons (Fig. 3a,b) and second the widely-used 3-(4,5-dimethylthiazol-2-yl)-2,5-diphenyltetrazolium bromide (MTT) assay and CellTiter-Blue (CTB) assays were performed on human SH-SY5Y neuroblastoma cells (Fig. 3c,d) providing two independent verifications of metabolic activity and cell viability.

The results revealed that oligomeric Aβ1-42 causes death of neurons while vAβ1-42 has no observable effects on cell health in any of the assays performed, even at high peptide concentrations. Primary neurons in culture are visibly affected with only a small population of healthy cells present at 3 days (Fig. 3a) and very few observable healthy cells after 7 days. In contrast, neurons treated with vAβ1-42 remain healthy over the time of the experiment (Fig. 3b). The MTT (Fig. 3c) and CTB (Fig. 3d) assays show a decrease in the metabolic activity and viabilty of neuroblastoma cells treated with Aβ1-42. vAβ1-42 has no effect on cell viability in any of the assays conducted and is comparable to vehicle buffer only.

### vAβ1-42 does not associate with primary hippocampal neurons

**Figure 4:**
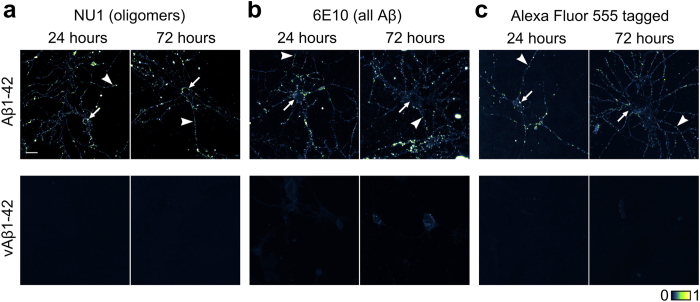
Hippocampal neurons treated with untagged oligomeric Aβ1-42 or vAβ1-42 (**a**,**b**) or Alexa fluor 555 tagged peptides (**c**) and imaged by confocal microscopy. Cells in a and b were fixed and stained with anti-oligomer antibody NU1^31^ (**a**) or 6E10 (**b**). Maximum projection images of six 0.5 μm slices are shown in a and c, b shows one 1 μm slice from the centre of a Z-stack. Scale bars 20 μm.

To provide additional insights into the behaviour of vAβ1-42 compared to Aβ1-42, we added each peptide (incubated for 2-4 hours) to primary rat hippocampal neurons and examined their distribution by two methods, immunolabelling with NU1 and 6E10 and using fluorescently tagged peptides (Fig. 4). The antibody labeling to Aβ1-42 is detected in the cell body and along the axons (Fig. 4a,b, top panels) and persisted for at least 3 days. In contrast, neurons treated with vAβ1-42 resemble the buffer only control (not shown) and show no labeling (bottom panels). Dot-blotting confirmed that 6E10 is able to bind to vAβ1-42 (Fig. 2d,e). Therefore, these results indicate that vAβ1-42 neither able to bind to the cell membrane nor be internalised and is removed from the cells after washing. To confirm that vAβ1-42 did not associate with the cells rather than being simply undetectable, a variation of this experiment was conducted using Alexa Fluor tagged versions of the Aβ peptides. This method has been used by our group previously and the fluorescent tag was shown not to affect assembly^27^. Cells treated with labeled versions of either Aβ1-42 or vAβ1-42 looked similar to those treated with antibodies, with only fluorescent Aβ1-42 present along neurites and around the cell body and no fluorescent signal for the vAβ1-42 treated neurons, even after three days. Whether antibodies were used or not, there was no evidence that vAβ1-42 either bound to the outside of the cell nor was internalised.

### vAβ1-42 has no effect on hippocampal synaptic function in primary hippocampal neurons

**Figure 5:**
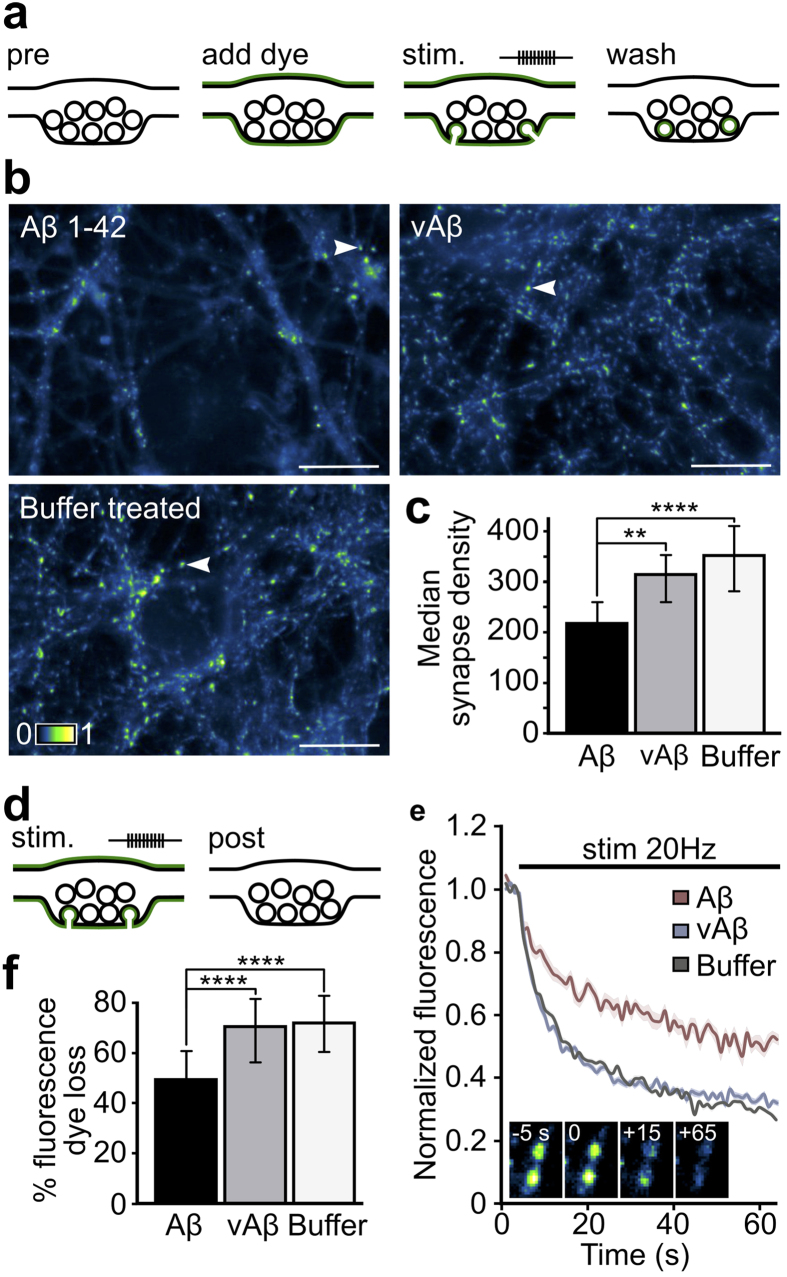
(**a**) Cartoon illustrating functional synaptic readout. Neurons are activated by field stimulation to evoke vesicle turnover in the presence of extracellular FM1-43-dye. Washing in dye-free solution leaves recently recycled vesicles fluorescently labelled. (**b**) Representative images of FM1-43 loading in neurons treated with Aβ1-42, vAβ1-42 or buffer. Arrowheads indicate discrete functional terminals. Scale bar 20 μm. (**c**) Histogram (median ± IQR) shows number of functional synapses expressed as the median synapse density per image, (Aβ1-42: 218 IQR 137-262, vAβ1-42: 315 IQR 258-355, buffer: 355 IQR 283–410, n = 20, 30, 30 images, respectively; Kruskal-Wallis one-way ANOVA, p = 0.0002 with Dunn’s multiple comparison test, see Methods for analysis). (**d**) Cartoon illustrating approach for readout of activity-induced dye-loss kinetics, corresponding to synaptic vesicle exocytosis. (***e***) Normalised fluorescence loss profiles for Aβ1-42, vAβ1-42 or buffer treated cells (average profiles of n = 218, 428, 560 synapses for Aβ1-42, vAβ1-42, buffer respectively). Shaded band denotes SEM for each trace. (***f***) Histogram of magnitude of FM1-43 destaining for data in (**e**), expressed as % dye loss (median ± IQR, Aβ1-42: 50 IQR 36-61, vAβ1-42: 71 IQR 56-82, buffer: 72 IQR 60-83, n = 218, 428, 560 synapses, respectively; Kruskal-Wallis one-way ANOVA, p < 0.0001 with Dunn’s multiple comparison test).

The function of small central synapses critically relies on the efficient fusion and retrieval of synaptic vesicles (SV). Aβ1-42 oligomers previously have been reported to modulate aspects of both recycling and vesicle pool organisation^33^. Here we used FM1-43, a widely employed fluorescent reporter of vesicle turnover to investigate the effects of Aβ1-42 and vAβ1-42 on vesicle recycling parameters^34^. In double-blind experiments, primary hippocampal neurons were treated 24 hours prior to imaging with a low concentration of 1 μM Aβ1-42, vAβ1-42, or buffer alone, and then subjected to field stimulation (1200 APs 20 Hz) in the presence of FM-dye to drive vesicle labelling (Fig. 5a). As a measure of synaptic vesicle recycling for the different treatments, representative images were collected after washing (Fig. 5b) and labelled terminals were quantified using a thresholding method (see Methods). Our analysis revealed that while vAβ1-42 and buffer alone had highly comparable synapse counts, the number of puncta in Aβ1-42-treated neurons was significantly lower (Fig. 5c, see figure legend for quantification). These results reveal that Aβ1-42 treatment leads to a significant deficiency in the recruitment of functional synapses compared to vAβ1-42 peptide or buffer alone conditions. Next, the consequences of the three incubation treatments on activity-driven FM1-43 dye-loss from labelled terminals (a measure of vesicle release) were examined (Fig. 5d). The level of dye-loss was highly comparable for vAβ1-42 and buffer alone conditions but was significantly less in Aβ1-42 treated neurons suggesting an impairment in exocytosis (Fig. 5e,f). Taken together, these findings indicate specific deficits in vesicle recycling properties in Aβ1-42 treated synapses, which are not observed in vAβ1-42 or buffer control treatments.

### Behavioural studies on an animal model (Lymnaea stagnalis, pond snail)

**Figure 6:**
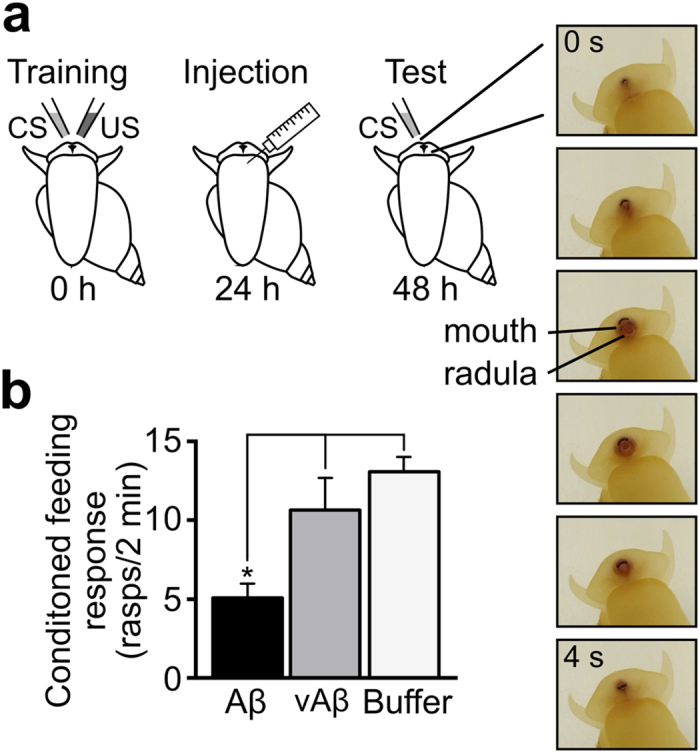
(**a**) Cartoon illustrating conditioned feeding response of *Lymnaea stagnalis* treated with Aβ1-42 or vAβ1-42. Animals were classically conditioned using the single-trial food-reward paradigm at 0 hours; injected with 1 μM Aβ1-42 (n = 55), 1 μM vAβ1-42 (n = 20), or vehicle (n = 106) at 24 hours; and tested for the conditioned feeding response at 48 hours. Picture inserts show an example of a complete feeding cycle (rasp) on which the behavioural assessment was based; rasping begins at 0 s, including opening of the mouth, protrusion of the toothed radula, ingestion of food, and closure of the mouth at 4 s. (**b**) One-way ANOVA, p < 0.0001. Tukey’s multiple comparison with p = 0.05: vAβ1-42 vs. Aβ1-42, Vehicle vs. Aβ1-42. Error bars are shown as ±SEM.

To investigate possible effects of vAβ1-42 on whole animal function, we took advantage of a well-characterised model system, the pond snail, Lymnaea stagnalis. This system has been used extensively for studies of both behavior and learning and memory, due to its ability to undergo robust classical conditioning and form stable long-term memories (for review, see ref. 18). Recent work has demonstrated that Aβ1-42 significantly impairs recall of long-term memory associated with single-trial food-reward classical conditioning^11^. Here, to investigate whether vAβ1-42 showed the same effect, vAβ1-42 was administered to snails. Briefly, animals were conditioned to associate a chemical stimulus with a food stimulus (Fig. 6a). Consolidated long-term memory can be tested by administering the chemical stimulus then measuring the animals’ feeding response^35^. For this experiment, animals were trained at 0 hours, injected with peptide or controls at 24 hours, and tested at 48 hours to determine if vAβ1-42 disrupts consolidated long-term memory (Fig. 6a). Comparison of the peptide effect on the conditioned feeding response following incubation with Aβ1-42 or vAβ1-42 with the vehicle (buffer only) control is shown in Fig. 6b. While Aβ1-42 treated animals display decreased conditioned feeding response to the chemical stimulus in comparison to vehicle treated animals, vAβ1-42 treated animals exhibit a similar conditioned feeding response to the vehicle control, suggesting vAβ1-42 does not disrupt recall of consolidated long-term memory. These findings support our cellular-level results and suggest that vAβ1-42 is largely inconsequential for neural network function.

Our results reveal a striking and important contrast between the activity of wild-type Aβ1-42 peptide and the novel variant vAβ1-42. Here we show that only the wild-type assembles to form A11 positive oligomers and amyloid fibrils. The Aβ1-42 oligomers are able to bind to and internalise into neuronal cells and show accompanying cellular toxicity. In stark contrast, the non-assembling vAβ1-42 is not associated with cell membranes, internalised and nor does it affect neuronal survival. We show that Aβ1-42 oligomers can impair synaptic vesicle recruitment and exocytosis at synapses. Finally, we were able to utilize the peptide in an animal behavior assay and reveal that whilst Aβ1-42 impairs memory recall, vAβ1-42 has no such effect. Only by comparison with this rationally-designed peptide variant can robust conclusions can be drawn and exciting new insights made into the effects of Aβ on neuronal cell function and animal behavior.

## Conclusions

Aβ is known to play an important role in the pathology of Alzheimer’s disease (AD), but the link between Aβ self-assembly and neurodegeneration remains elusive. It is clear however that the conformational change that accompanies assembly is a critical factor underlying amyloidogenic protein toxic action. To investigate this further, we rationally designed a non-assembling peptide variant of Aβ1-42 that shares size and sequence with Aβ but does not self-assemble into oligomers or fibres. The identification of such a peptide is an essential contribution to the field and provides a powerful tool for elucidating structure-toxicity relationships and rigorously assessing the mechanisms of Aβ induced neuronal dysfunction in cellular assays and animal models.

Here, we have examined the potential amyloidogenic regions of Aβ1-42 and rationally designed a peptide variant that does not aggregate over the course of one week, does not cause toxicity in cell culture or behavioural effects in an animal model, nor binds to or is taken up by neuronal cells in culture. The double substitutions, F19S and G37D completely removed amyloidogenicity and the variant peptide was confirmed experimentally as assembly-incompetent. Several proof-of principle examples of the rational design strategy are provided, and show that self-assembly is key to Aβ1-42 detrimental effects.

Modelling the neurotoxic properties of Aβ1-42 using cells in culture is challenging but critical if its pathogenic mechanisms are to be understood at a molecular level. In AD, it is likely that a range of Aβ1-42 aggregates are produced that may all contribute to neuronal death and it may not be the case that one species in particular (e.g. dimer, trimer, octomer) is responsible. We sought to replicate this environment as closely as possible, carrying out our functional assays with a heterogeneous Aβ1-42 oligomer preparation in order to simulate how the dynamic nature of amyloid assembly, as well as the assemblies themselves, might be detrimental to cells. To this end, a suitable peptide that is unable to self-assemble is essential since it is not possible to produce a stable monomer of wild-type Aβ1-42.

It is paramount that peptides, and not just buffers or vehicle solutions, are used to compare with wild-type Aβ in AD research. In our experiments, vAβ1-42 has the same effect as buffer controls, supporting observations that aggregated, and not monomeric isoforms of Aβ1-42 are toxic. These conclusions can only be drawn by comparison of Aβ1-42 with another peptide similar in sequence and, crucially, that has undergone the same preparation method. Solvents such as HFIP are often used to solubilise the peptide and can produce false positive results if not completely removed. By preparing vAβ1-42 using the procedure described here, any contaminating effects would be uncovered.

Our data show that vAβ1-42 remains in a soluble random coil conformation throughout the course of the experiment and it is likely that for this reason its detrimental effects are abrogated. The structure of Aβ1-42 oligomers has been shown to play a very important role in their toxic function^5^,^6^,^8^,^32^ and these results confirm that oligomerisation is required for Aβ1-42 to associate with neurons, affect neuronal viability and exert behavioural effects.

The data described here outlines an unprecedented characterisation of a novel peptide and show unambiguously in several models that self-assembly is required for neurotoxicity. This novel peptide allows new insight into the pathway of Aβ action, revealing that β-sheet rich, oligomeric Aβ1-42 accumulates within neuronal cells. Our results show that it is likely that the oligomeric structure is crucial for membrane binding and internalisation. Once inside, it is able to exert detrimental effects on synaptic function in hippocampal neurons and on long-term memory in a model organism. In contrast, the non-assembling vAβ1-42 lacks the ability to form β-sheet rich oligomers and is unable to exert any of these effects, providing strong evidence that self-assembly is fundamental for the damaging effects of Aβ.

## Materials and Methods

### Peptide design

Sequence based design was performed using the WALTZ algorithm^19^ to explore the effect of amino-acid substitutions on the predicted amyloidogenicity of the Aβ1-42 peptide. The graph produced using WALTZ shows two peaks that indicate the location of two amyloidogenic regions (residues 16–21 and residues 37–42) in the wildtype Aβ1-42 peptide. Substitutions were introduced within the predicted amyloidogenic regions to examine the effect on the graphical output prediction. All possible substitutions at positions 19 and 37 were investigated and ranked based on their ability to reduce the amyloidogenicity peak. A number of variants were shown to reduce the predicted amyloidogenicity and two were selected based on previous experimental assembly studies.

### Preparation of peptides

Hexafluoroisopropanol (HFIP) films of recombinant Aβ1-42 were purchased from rPeptide and vAβ1-42 was purchased from JPT. Peptides were prepared using an adapted protocol originally developed in^17^. HEPES buffer (10 mM HEPES, 50 mM NaCl, 1.6 mM KCl, 2 mM MgCl2, 3.5 mM CaCl2) was used to mimic the culture media as previously described^3^,^17^,^27^. All preparation was conducted using protein LoBind Eppendorfs and tips (Alpha Laboratories). Briefly, 0.2 mg Aβ1-42 (rPeptide) was solubilised in 200 μL HFIP (Sigma-Aldrich) to disaggregate the peptide. The solution was then vortexed on high for one minute and sonicated in a 50/60 Hz bath sonicator for five minutes. HFIP was dried completely using a low stream of nitrogen gas. Once completely dried, 200 μL dry dimethylsulfoxide (DMSO) (Sigma-Aldrich) was added, vortexed for one minute, and sonicated for one minute. Solutions were added to a 7K MWCO Zeba buffer-exchange column (Thermo Scientific) equilibrated with HEPES buffer with 40 μL HEPES as a stacking buffer. The protein solution was kept on ice and the absorbance at 280 nm measured with a NanoDrop spectrophotometer using a molar absorption coefficient of 1490 M^−1^ cm^−1^. Solutions were immediately diluted to 50 μM with HEPES buffer and incubated where indicated or used immediately in further experiments.

### Generation of Alexa Fluor 555-conjugated peptides

Peptides were treated as outlined above up to the addition of 200 μl dry DMSO. The Alexa Fluor 555 tag was prepared as per manufacturers instructions (Life Technologies). Briefly, 10 μl H_2_O was added to the Alexa Fluor TFP ester and kept on ice. This was added to the peptide in 200 μl DMSO along with 10 μl 1 M sodium bicarbonate pH 8.3, mixed and incubated for 15 minutes at room temperature protected from light. Following this the procedure resumed as above and the solution added to the Zeba buffer exchange column. The calculations for the concentration was adjusted to take into account the absorbance of the dye.

### Transmission electron microscopy

4 μl of 50 μM Aβ was placed on the surface of Formvar/carbon film coated, 400 mesh copper grids (Agar Scientific) and allowed to absorb for two minutes and blotted dry. A 4 μl aliquot of milliQ-filtered water was then added to the grid and blotted. Immediately after this the grid was negatively stained with 4 μl of 2% (w/v) uranyl acetate for 2 minutes, blotted and dried. The uranyl acetate wash was repeated once more and the grid was left to air dry. All TEM grids were examined using a Hitachi-7100 TEM at 100 kV and the images were acquired digitally with an axially mounted (2000 × 2000 pixel) Gatan Ultrascan 1000 CCD camera. Aliquots of Aβ peptide samples were taken at different time points to monitor the fibrillation state and morphology.

### Circular dichroism

300 μl of 50 μM of Aβ peptide (prepared as described above) in phosphate buffer (pH7.4) was placed into a 1 mm path length quartz cuvette (Hellma) and scanned between 180 nm and 275 nm on a JASCO J715 Spectropolarimeter. The samples were equilibrated at 20 °C using a water bath. Three spectra were averaged for each measurement.

### Thioflavin T fluorescence

10 μM of ThT in 50 μM Aβ peptide was added to a 10 mm cuvette. An emission scan between wavelength 460–600 nm was performed in a Varian Cary Eclipse Fluorescence Spectrophotometer. The sample compartment temperature was set to 21 °C, scan rate was 600 nm/min and three spectra were averaged for each measurement.

### Dot blotting

5 μl of 50 μM Aβ peptide was spotted onto a nitrocellulose membrane and allowed to dry. The membrane was boiled with PBS for three minutes twice and then incubated at room temperature with blocking buffer (10% milk in 0.1% TBS-T) for 1 hour. The blocking buffer was poured off the membrane and replaced with primary antibody overnight at 4 °C. The membrane was washed for 10 minutes three times with 1% TBS-T before being incubated with HRP conjugated secondary antibody for 30 minutes. Following more washes the membrane was incubated with ECL substrate (Millipore) for three minutes before being developed. Antibodies were purchased from Cambridge Bioscience (6E10 and 4G8). NU1 was a gift from the William Klein lab^31^.

### Western Blotting

2 μg of peptide in 4x Laemelli Sample Buffer (Bio-Rad) were loaded on a 4–20% Mini-PROTEAN TGX Stain-Free gel (Bio-Rad) and run in 1X running buffer (diluted from 10X stock of 25 mM Tris Base, 192 mM Glycine, 1% SDS) at 100V. The gel was then transferred in 1X transfer buffer (10% 10X stock (25 mM Tris Base, 192 mM Glycine), 10% Methanol, 80% ddH20) at 25V for 2 hours on to nitrocellulose 0.45 μm membrane. The membrane was blocked with 10% Milk/TBS-T at room temperature for 1 hour then incubated with a 1:10000 dilution of 6E10 antibody overnight at 4 °C. The membrane was washed three times for ten minutes with 1% TBS-T before being incubated with a 1:10000 dilution of HRP conjugated anti-mouse secondary antibody at room temperature for 30 minutes. The membrane was washed again three time for ten minutes with 1% TBS-T and incubated with ECL substrate for five minutes before being developed.

### Nuclear magnetic resonance

vAβ1-42 was prepared at 200 μM concentration in 10% v/v D_2_O standard. 1H NMR spectra (128 scans) were acquired every 30 minutes over a period of 66 hours. The temperature was regulated at 25 °C. The spectra were processed with 1.5 Hz line broadening prior to base line correction and Fourier transformation. The residual solvent signal around 4.8 ppm was cut for clarity.

### Cell culture

SH-SY5Y cells were cultured as described previously^27^ in Dulbecco’s modified Eagle medium (DMEM) supplemented with 10% Foetal Calf Serum, 1% Penicillin-Streptomycin and 2 mM L-Glutamine. Cells were passed every 4–5 days at around 80% confluency and not used beyond passage 19.

Rats are housed within a specialised facility under Home office guidelines and sacrificed using procedures in accordance with Animals (Scientific Procedures) Act 1986, Amendment Regulations 2012. Primary neurons were prepared from the hippocampus of P0-P1 rats by initially dissecting the tissue into ice cold HBSS containing 0.1 M HEPES. Following washes in pre-warmed Basal Medium Eagle (BME) (Gibco) containing 0.5% glucose, 2% FCS, 1 mM Na-Pyruvate, 0.01 M HEPES pH 7.35, 1% Penicillin-Streptomycin, 1% B27 supplement and 1% Glutamax, tissues were triturated using a 1 ml pipette until fully dissociated. The cell suspension was diluted further with complete BME media and approximately 40,000 cells plated into 2 cm^2^ wells containing a coverslip coated in 20 μg/ml Poly-D-Lysine with a layer of hippocampal astrocytes that had been growing for 4–5 days. After 2–3 days cells were treated with 3.25 μM cytosine arabinoside to curb further proliferation of astrocytes. Cells were used 10–14 days after plating.

### Cell viability assay with primary hippocampal neurons

After the desired incubation time, one drop of each Readyprobes reagent (Life Technologies) was added per well. The kit contains a blue stain to label all cells, and a green stain to label dead cells only. Cells were incubated for 15 minutes then imaged on a Zeiss CO widefield microscope using DAPI and FITC filters. Images were analysed using FIJI software as follows. Regions of interest were drawn around neuronal cell bodies (astrocytes were excluded) using DIC and DAPI channels, which indicated total cell number (live and dead). Background values in the green channel (dead cells) were determined and any value greater than this was ascribed as positive i.e. dead. Numbers of dead cells are expressed as a percentage of the total number of cells. Between nine and seventeen regions in total per sample were imaged from either two or three coverslips from experiments performed on three separate occasions using newly prepared peptide.

### Cell metabolism assays with neuroblastoma cells

The Vybrant MTT [3-(4,5-dimethylthiazol-2-yl)-2,5-diphenyl-2H-tetrazolium bromide] cell-proliferation assay (Invitrogen) was used on undifferentiated SH-SY5Y cells according to the manufacturer’s protocol. Briefly, SH-SY5Y cells (10^−4^ cells/well) were seeded on a 96-well plate 2 days prior to the assay. The cells were then incubated with 10 μM oligomeric Aβ1-42 or vAβ1-42 for 24 or 48 hours at 37 °C. At these time points, 12 mM MTT solution was added to the cells and further incubated for 4 hours at 37 °C. The resulting insoluble dye was dissolved with 50 μL of DMSO and the absorbance measured at 540 nm with a 620 nm reference filter. For CTB (Cell-Titer Blue), cells were plated as above except 2 hours before treatment with peptide, the media was replaced with serum-free media. After 24 or 48 hours, 20 μL of CTB dye was added to each well and further incubated for 6 hours. Fluorescence was measured at 570 nm. Experiments were carried out in triplicate a minimum of three times and the data pooled. Background absorbance or fluorescence was calculated from a dead cell control. Triplicate values were averaged then subtracted from sample values. This was carried out separately for each time point in each experiment. Statistical analysis was carried out using GraphPad Prism software.

### Immunofluorescent labeling and confocal microscopy

Primary neuronal cells were treated with 1 μM of either Aβ1-42 or vAβ1-42 oligomers, or volume-matched buffer, for 24 and 72 hours. For 6E10 staining, 1 μM Aβ1-42 or 5 μM vAβ1-42 were added. After removal of media, cells were immediately fixed with 2% paraformaldehyde for 15 minutes. Following a wash with wash buffer (25% Superblock (Thermo Fisher) in PBS), cells were permeabilised with 0.3% Triton-X 100 for 10 minutes then 50 mM glycine added to block unreacted aldehydes. Cells were incubated with Image-IT signal enhancer (Life Technologies) for 30 minutes then cells were blocked using undiluted Superblock for 30 minutes. Primary antibody NU1^31^ or 6E10 (Cambridge Biosciences) (both 1:500) diluted in wash buffer was incubated with the cells for one hour. After washing, Alexa Fluor 555 (6E10) or 488 (NU1) conjugated goat anti-mouse secondary antibody (1:500) (Life Technologies) was added for the same period and after a final wash the coverslips were mounted in ProLong Gold (Life Technologies), cured for 2 days then imaged. Cells were imaged using a 63 × 1.2 NA water objective (tagged peptide or NU1 antibody) or a 40 × 1.1 NA water objective (6E10 antibody) on a Leica SP8 confocal microscope. Emission from NU1 stained cells (488 secondary) was collected using a 488 nm excitation laser line between 495 and 540 nm on a PMT detector. Emission from Alexa Fluor-555 tagged peptide treated cells and 6E10 stained cells (555 secondary) was collected using a 561 nm laser line between 555 and 650 nm. Samples were imaged sequentially.

For cells treated with tagged peptides, the cells were fixed, washed and mounted as described above.

### Assessments of synaptic function

10 days *in vitro* primary hippocampal neurons grown on coverslips were incubated for 24 h with 1 μM Aβ 1-42, vAβ1-42 or buffer control. For imaging experiments, a neuronal culture was transferred into external bath solution (in mM: 37 NaCl, 5 KCl, 2.5 CaCl2, 1 MgCl2, 10 D-glucose, 5 HEPES, 20 μM 6-cyano-7-nitroquinoxaline-2,3-dione and 50 μM d(–)-2-amino-5-phosphonovaleric acid) and field stimulation (1200 APs at 20 Hz) applied in the presence of FM1-43 (10 μM) dye to label synapses. After completion of endocytosis, residual surface fluorescence was removed by washing in dye-free saline. The experimenter was blind to the treatment protocol for each coverslip during both imaging and data analysis. All analysis was carried out on non-filtered, raw images in ImageJ. For the quantification of functional synapses ten regions were sampled for each coverslip and converted to maximum intensity projections based on 4 × 0.5 µm image stacks. Terminals were identified using objective automated methods based on isodata thresholding (IJ_IsoData core function, ImageJ, NIH, USA). For activity-evoked dye-loss analysis, we applied a further destaining protocol (1200 APs 20 Hz) to FM1-43 dye-loaded coverslips while imaging. Destaining synapses were identified blindly on the basis of subtracted images before and after the destaining stimulus, and ROIs of equal size (2.1 × 2.1 µm) were drawn around fluorescent puncta. Background fluorescence was subtracted from each ROI and the destaining curves were normalised to the average value of five points before the onset of the stimulation. The % destaining was calculated using the average of the last five frames from the end of the stimulation. Images were collected on an Olympus BX61WI microscope equipped with a x60 1.0 N.A. dipping objective, excitation and emission filter sets at 480/20 and 520/35, and an Olympus XM10 camera with 2 × 2 binning. Statistical analysis was carried out in GraphPad Prism using Kruskal-Wallis one-way ANOVA followed by Dunn’s multiple comparison test.

### Comparison of the effect of the peptides on memory in Lymnaea stagnalis

Pond snails, Lymnaea stagnalis, were bred at the University of Sussex and maintained in large holding tanks containing 18–22 °C copper-free water, at a 12:12 hour light-dark cycle. The animals were fed Tetra-Phyll (TETRA Werke) twice a week and lettuce three times a week.

The peptides were administered to the animals directly after preparation. Using a 1 mL syringe with 30 gauge precision glide needles (Becton Dickinson), 100 μL of the Aβ1-42 or vAβ1-42 was injected into the haemolymph (~1 μL in volume) of each snail. The estimated final concentration in the animal was 0.1 μM for Aβ1-42 and vAβ1-42. As there is no blood-brain barrier in Lymnaea^36^, the injected peptides have direct access to the animal’s central nervous system. For vehicle-injected control animals, 100 μL of normal saline was injected.

Using well-established methods^37^, four-to six-month-old snails were removed from their home tanks and starved in new tanks for two days at the same temperature and light dark cycle as the home tanks. After the starvation period, the animals underwent single-trial food-reward classical conditioning^35^ in which the conditioned stimulus (CS) (amyl acetate: 0.004% final concentration) and the unconditioned stimulus (US) (sucrose: 0.6% final concentration) were paired. Initially, each individual snail was left to acclimatise in a 14 cm diameter Petri dish with 90 mL of 18–22 °C copper-free water for ten minutes. After the acclimatisation period, 5 mL of amyl acetate was added to the dish and after thirty seconds, 5 mL of sucrose was added. The snails were then left in their Petri dishes for two minutes, and then removed to their starvation tanks. Both the vehicle-injected and Aβ-injected groups were trained.

All animals were tested with the CS. Each individual snail was left to acclimatise in a 14 cm-diameter Petri dish with 90 mL of 18–22 °C copper-free water for ten minutes. After the acclimatisation period, 5 mL of 18–22 °C copper-free water was added to the dish. Rasps, the animals’ feeding movements, were manually counted for two minutes to determine a baseline rasping rate (number of rasps per two minutes) for each individual. After two minutes, 5 mL amyl acetate was added to the dish. Rasping was tracked for two minutes. Rasping rates were determined by subtracting the individual animal’s baseline rasp from the amyl acetate induced rasp.

Data that passed the D’Agostino and Pearson omnibus normality test were subjected to parametric tests (one-way analysis of single variance [ANOVA] with Tukey’s multiple comparison) to establish significance (criterion, p < 0.05). GraphPad Prism software was used for all analyses.

## Additional Information

**How to cite this article**: Marshall, K. E. *et al*. A critical role for the self-assembly of Amyloid-β1-42 in neurodegeneration. *Sci. Rep.* **6**, 30182; doi: 10.1038/srep30182 (2016).

## Supplementary Material

Supplementary Information
