# Supplementary material for: A critical role for the self-assembly of Amyloid-β1-42 in neurodegeneration
Source: Sci Rep. 2016 Jul 22;6:30182. doi: 10.1038/srep30182 (PMC4957119; doi:10.1038/srep30182)
Supplement: Supplementary Information [file srep30182-s1.pdf]

A critical role for the self-assembly of Amyloid-β1-42 in neurodegeneration

Karen E Marshall[1], Devkee M Vadukul[1], Liza Dahal[1,2], Alina Theisen[1,3], Milena W Fowler[1], Youssra Al-Hilaly[1,6], Lenzie Ford [1,4,5], György Kemenes[1], Iain J Day[1], Kevin Staras[1], Louise C Serpell[1*]

[1] School of Life Sciences, University of Sussex, Falmer, BN1 9QG, UK

[2] Current address: Department of Chemistry, University of Cambridge, Lensfield road, Cambridge, UK

[3] Current address: Manchester Institute of Biotechnology, University of Manchester, 131 Princess Street, Manchester, M1 7DN, UK

[4] Current address: Department of Neuroscience, Columbia University, 1051 Riverside Drive, New York, NY 10032, USA

[5] Current address: Howard Hughes Medical Institute, Columbia University, 1051 Riverside Drive, New York, NY 10032, USA

[6] College of Sciences, Chemistry Department, Al-Mustansiriyah University, Baghdad, Iraq

Supplementary information

Methods

Nuclear magnetic resonance

vAβ1–42 was prepared at 200 μM concentration in 10% v/v D_2O standard. ^1H NMR spectra (128 scans, 599.6 MHz ^1H frequency) were acquired every 30 mins over a period of 66 hours. Solvent suppression was provided by a double pulsed field gradient spin echo method (Hwang et al., 1995). The temperature was regulated at 25 °C. The spectra were processed with 1.5 Hz line broadening prior to base

line correction and Fourier transformation. The residual solvent signal around 4.8 ppm was cut for clarity.

Supplementary figure 1.

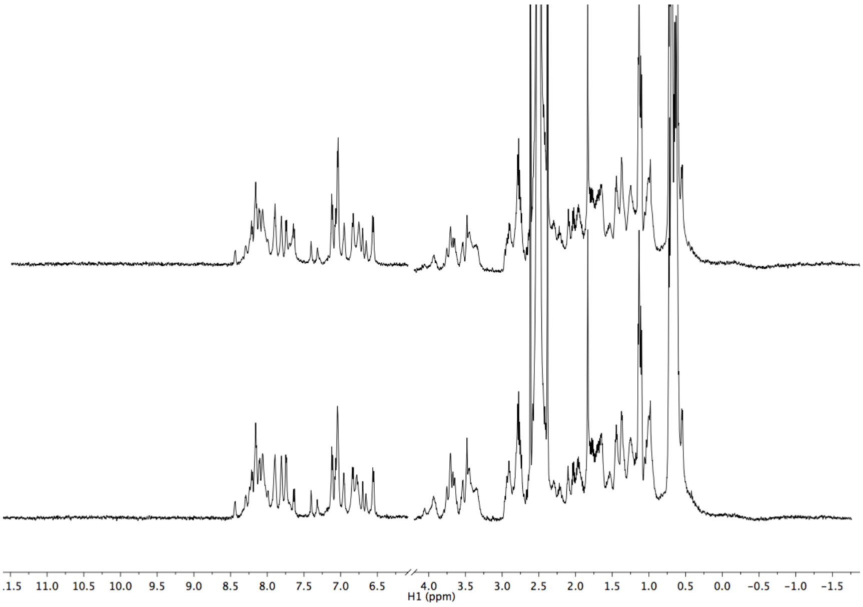

Solution-state NMR spectra of vAβ1–42 at 0h and after 66h. vAβ1–42 was prepared at a concentration of 200 μM and scans taken every 30 minutes (time points not shown). The initial spectrum and final spectrum remained identical in shift positions and signal strength confirming that the vAβ1–42 retains solubility and a constant structure.

Reference

T. L. Hwang and A. J. Shaka, J. Magn. Reson. A. 112 (1995) 275-279
